# Multifocal Carcinoid Tumor of Small Intestine: A Rare Cause of Chronic Obscure Gastrointestinal Bleeding, Suspected on Capsule Endoscopy and Diagnosed on Double Balloon Enteroscopy

**DOI:** 10.4021/gr303w

**Published:** 2011-05-20

**Authors:** Jose C. Hernandez, Juan Rojas, Suryakanth R. Gurudu

**Affiliations:** a Division of Gastroenterology and Hepatology, Mayo Clinic-AZ, 13400 East Shea Boulevard, Scottsdale, Arizona, USA

**Keywords:** Carcinoid tumor, Video capsule endoscopy, Double-balloon enteroscopy

## Abstract

We reported a case of multifocal carcinoid tumor of small intestine causing chronic obscure gastrointestinal bleeding, suspected on capsule endoscopy and diagnosed on double balloon enteroscopy.

## Case Report

A 64-year-old man with bouts of obscure, overt gastrointestinal bleeding (OGIB) in the past was transferred to our medical center for persistent melena. Over the past five years, he had undergone extensive inpatient evaluations at several hospitals for OGIB without a cause identified. On this occasion, the patient was found to have a hemoglobin of 5.7. He underwent a push enteroscopy which showed no obvious bleeding source and colonoscopy which showed evidence of old hemorrhage. A repeat video capsule endoscopy (VCE; Pill Cam SB, GIVEN, Yokneam, Israel) was performed which showed several polyploid lesions in the mid small bowel and continuing through the terminal ileum with fresh blood noted (see video).

**Figure 1:**
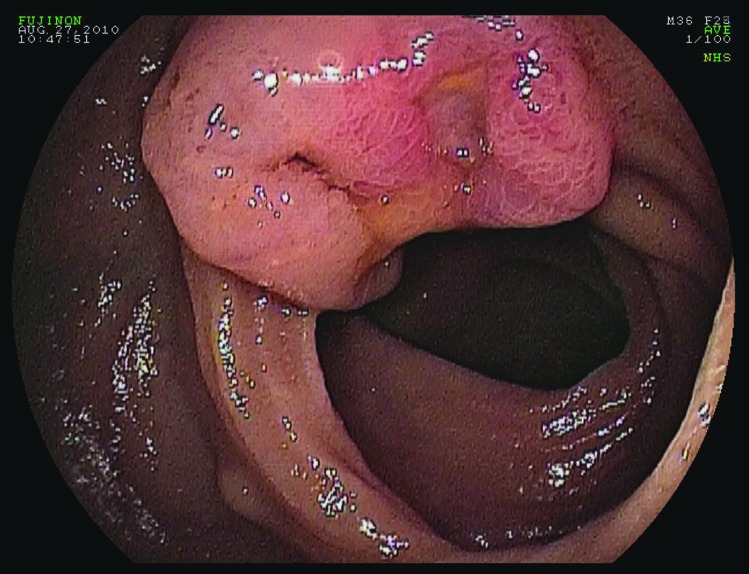
Double-balloon enteroscopy. Friable, ulcerated sessile polypoid lesion in the mid ileum.

**Figure 2:**
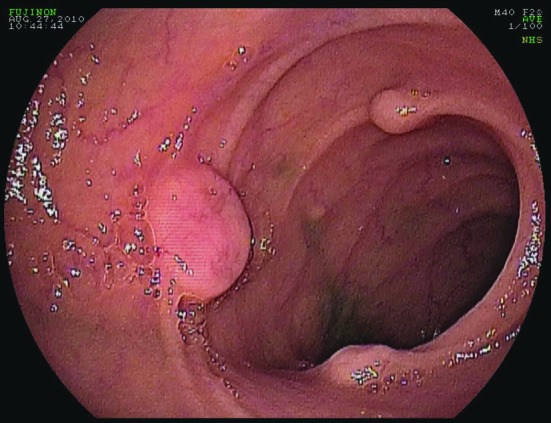
Double-balloon enteroscopy. Multiple small polypoid lesions in the distal ileum.

**Figure 3:**
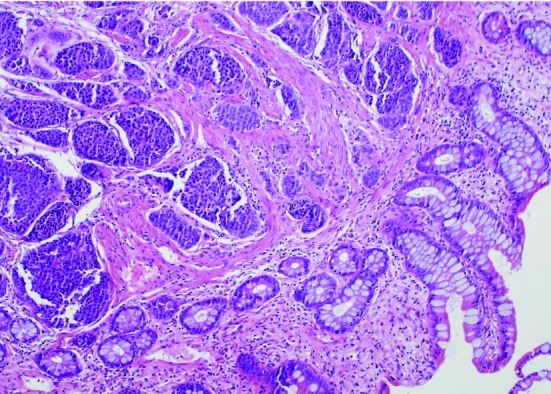
Histopathology. Biopsies from distal ileum polyps showed low-grade neuroendocrine pathology consistent with carcinoid tumor (hematoxylin and eosin stain; magnification x 400).

Following transfusion with 8 units of packed red blood cells, he was transferred to our facility for retrograde double-balloon enteroscopy (DBE). His study was significant for two large, friable, ulcerated sessile polypoid lesions in the mid ileum (Fig. 1) and multiple small polypoid lesions in the distal ileum (Fig. 2). Biopsies taken from these polyps showed low-grade neuroendocrine pathology consistent with carcinoid tumor (Fig. 3). Further investigations performed at our medical center included a CT enterography and octreotide scan which confirmed localization of the tumor to the distal small bowel. He later underwent successful laparoscopic partial small bowel resection.

## Discussion

Carcinoid tumors are a form of neuroendocrine tumors and can present a dilemma for clinicians because the disease can encompass a wide range of clinical behaviors. Midgut carcinoids are the most common form of carcinoid tumor and originate most frequently in the terminal ileum. Due to the indolent behavior of small bowel carcinoid tumors, patients usually have symptoms for a mean of 5 years before the diagnosis is made [1]. Carcinoid syndrome occurs in only approximately 20% of patients with small bowel carcinoid.

Recent literature suggests that the diagnostic yield of DBE for suspected primary small bowel neuroendocrine tumor is low [2]. As illustrated by a recently published database, the most common indication for DBE that ultimately led to the diagnosis of small bowel carcinoid included suspected small bowel malignancy, obstructive symptoms, and OGIB [3]. As described in our case and in other published studies, DBE commonly detects small bowel mass lesions responsible for OGIB that are commonly missed or not readily identified by VCE [4]. Multiple submucosal masses presenting as chronic melena and ultimately diagnosed as multifocal carcinoid tumor in the proximal ileum have been described. To our knowledge, this is the first reported case that utilized both VCE and DBE in rapid succession to attain tissue diagnosis of small bowel carcinoid tumor in a non-invasive fashion. Our case illustrates that further studies combining the two methods during the evaluation of OGIB may serve to increase the diagnostic yield.
